# A novel mutation in the *PEX26* gene in a family from Dagestan with members affected by Zellweger spectrum disorder

**DOI:** 10.1016/j.ymgmr.2021.100754

**Published:** 2021-04-12

**Authors:** Natalia A. Semenova, Marina V. Kurkina, Andrey V. Marakhonov, Elena L. Dadali, Natalia N. Taran, Tatyana V. Strokova

**Affiliations:** a Research Centre for Medical Genetics, 1 Moskvorechye Street, Moscow 115522, Russian Federation; b Federal Research Centre of Nutrition and Biotechnology, Kashirskoe shosse, d. 21, Moscow 115446, Russian Federation

**Keywords:** Zellweger syndrome spectrum, *PEX26* gene, Cholestasis, Hepatic dysfunction, ALT, alanine aminotransferase, AST, aspartate aminotransferase, CI, confidence interval, GGT, gamma-glutamyltranspeptidase, DBS, dried blood spot, LDG, lactate dehydrogenase, OMIM, Online Mendelian Inheritance in Man, PBD, peroxisome biogenesis disorders, VLCFA, very-long-chain fatty acids, ZSD, Zellweger spectrum disorders

## Abstract

**Background:**

Peroxisome biogenesis disorders (PBD) are a heterogeneous group of autosomal recessive disorders that affect multiple organ systems. Approximately 80% of PBD patients are classifiedin the Zellweger syndrome spectrum, which is generally caused by mutations in the *PEX1, PEX6, PEX10, PEX12*, or *PEX26* genes.

**Methods:**

We present the clinical characteristics of three male members with cholestatic hepatopathy and developmental delay. Next-Generation Sequencing (NGS) was used to analyze 52 genes responsible for hereditary diseases with cholestasis. The variant was confirmed by Sanger sequencing. Dried blood spot (DBS) samples of 537 newborns from Dagestan were tested for the presence of that mutation. The frequency of the mutant allele in the population of Dagestan wasestimated using the Hardy–Weinberg equilibrium.

**Results:**

Symptoms of disease manifested from the first months of life as severe hepatic dysfunction and developmental delay. Physical examination showed jaundice, hepatosplenomegaly, coagulopathy, and normal or slightly elevated level of gamma-glutamyltransferase (GGT), similar to progressive familial intrahepatic cholestasis. The level of C26 and ratio of C26/C22 in plasma were increased. A nucleotide variant in the *PEX26* gene was identified: NM_017929.6:c.347 T>A, p.(Leu116Gln) in a homozygous state. Parents and healthy siblings were heterozygous for the mutant allele. This variant was not described in the Database of Single Nucleotide Polymorphism (dbSNP), it is not registered in the Human Gene Mutation Database (HGMD) v. 2020.1. The frequency of the mutant allele in the population of Dagestan is estimated to be less than 0.000931 (99% CI, 0.000929–0.000934).

**Conclusions:**

Our clinical cases from Dagestan describe the phenotype associated with the c.347 T>A,p.(Leu116Gln), variant in the *PEX26* gene. We show that the onset of the clinical picture in patients with Zellweger syndrome spectrum could start with severe hepatic dysfunction and cholestasis. We suggest that biochemical screening of PBD in infants with cholestasis is necessary.

## 1 Background

Peroxisomal disorders are generally divided into peroxisome biogenesis disorders (PBD) and peroxisomal single enzyme deficiencies. Zellweger syndrome (ZS), the prototype of PBD, was first described in 1964 [1]. To date, 27 genetics forms of ZS are described in OMIM database (PS214100). Typically, affected children present with multiple problems of different organs including severe neurological abnormalities (hypotonia, developmental delay, epilepsy), skeletal abnormalities (rhizomelia, abnormal calcification), hepatic dysfunction (hepatomegaly, abnormal liver function), and typical facial dysmorphism [2]. In addition to classical ZS, milder forms of ZS are collectively referred to as Zellweger spectrum disorders (OMIM# 601539) [2] and include neonatal adrenoleukodystrophy and Infantile Refsum Disease (IRD). ZSD has an overall incidence of 1:50,000 births in the United States [3,4]. During recent years, the understanding of peroxisomal structure and function of specific peroxins has been achieved [5,6]. The main function of peroxisomal metabolism is the β-oxidation of very-long-chain fatty acids (VLCFA) [7]. The elevation of VLCFA in plasma has been demonstrated in patients with Zellweger syndrome. Peroxisome biogenesis disorders are caused by mutations leading to impaired function of peroxins, which are proteins responsible for peroxisome assembly and biogenesis [8]. Biallelic mutations in the *PEX1* (OMIM# 602136), *PEX6* (OMIM# 601498), *PEX10* (OMIM#602859), *PEX12* (OMIM# 601758), or *PEX26* (OMIM# 608666) genes are found in approximately 90% of ZSD patients. The *PEX1* and *PEX6* genes are most commonly affected among patients with a PBD with frequencies of 60 and 16%, respectively [9]. The *PEX26* gene encodes the peroxisome assembly factor termed peroxin Pex26, which is involved in peroxisomal matrix protein import [10]. Homozygous or compound-heterozygous pathogenic variants in the *PEX26* gene cause ZSD of complementation group 8, which have variable clinical manifestations ranging from a severe ZS (PBD7A; OMIM# 615872) to a less severe IRD (PBD7B; OMIM# 614873). Here we describe a family with three affected individuals who are all homozygous for a missense variant in *PEX26* and the manifestation of the disease in first months of life, with severe hepatic dysfunction similar to progressive familial intrahepatic cholestasis.

## 2 Methods

A family with three affected male members were clinically examined at the Research Centre for Medical Genetics, Moscow, Russia. The study was approved by the local ethics committee of the Research Centre for Medical Genetics (the approval number 2018-1/3).

### 2.1 Biochemical analysis

The Folch extraction method was used to harvest VLCFA, phytanic acid, and pristanic acid from plasma/serum (Folch). All lipid fractions were derivatized (methylated) for GC–MS with boron trifluoride-methanol solution (10%) according to manufacturer's recommendations.

VLCFA, phytanic acid, and pristanic acid were analyzed by GC/MS 7890А/5975С (Agilent Technologies, USA) with НР-5МS. The flow rate was 1 ml/min and the oven temperature was 40 °C for 1 min, increasing to 280 °C at 10 °C/min, then held for 10 min. The temperatures were 260°С, 260°С, 250°С for the injection port, transfer line and ion source respectively. The methyl esters of fatty acids in hexane were injected by an automatic sampler G4567A (Agilent Technologies, USA). The fatty acids were identified by their retention time and mass spectrum. These values were compared with commercial standarts (Sigma). Phytanic and pristanic acids were analyzed in SIM-mode.

### 2.2 DNA analysis

Genomic DNA was extracted from whole blood with EDTA using GeneJET Genomic DNA Purification Kit (Thermo Fisher Scientific, USA). Direct sequencing was performed on ABI PRISM 3500xL Genetic Analyzer (Applied Biosystems, USA). Massive parallel sequencing was performed with Ampliseq technology on Ion S5 (Thermo Fisher Scientific, USA).

For the patient P –IV/2 we used the panel targeting coding exons of 56 genes which associated with inherited diseases with cholestasis and including the 6 PEX genes (*PEX1, PEX10, PEX12, PEX26, PEX6, PEX7***)**. For the other patient (P-IV/4) we performed targeted sequencing of 587 genes associated with metabolic disorders including 14 PEX genes (*PEX1, PEX10, PEX11B, PEX12, PEX13, PEX14, PEX16, PEX19, PEX2, PEX26, PEX3, PEX5, PEX6, PEX7*). Nucleotide variants were initially prioritized by minor allele frequency <2% in the general population and selected based on clinical diagnosis using human phenotype ontology (HPO) terms. Sanger sequencing was used to verify the mutation in affected individuals and family members.

### 2.3 Population analysis

We tested dried DBS samples of 537 newborns from Dagestan for the presence of that mutationby PCR-RFLP. Using the Hardy–Weinberg equilibrium, we estimated the frequency of the mutant allele.

## 3 Results

### 3.1 Clinical characteristics

**Fig. 1:**
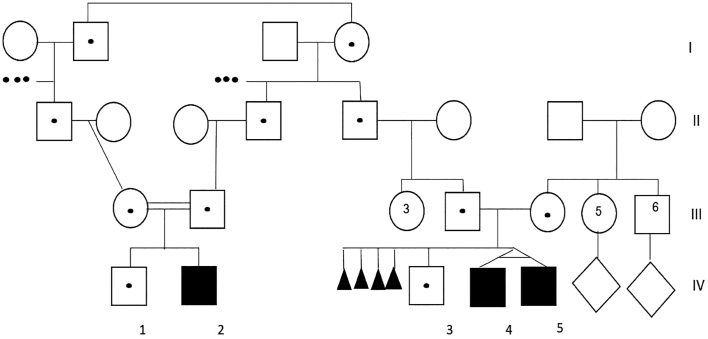
Family pedigree with the tree of affected members 2, 4, and 5 in the fourth generation.

We present a family from Dagestan with three male members of the first year of life with cholestasis hepatopathy and developmental delay (Fig. 1).

Patient IV/2 (P–IV/2). Male patient, 7 months old. Delivery was at 39 weeks of gestation with a weight of 3050 g (50th cent) and length of 50 cm (50th cent). The APGAR score was 8/9. The perinatal period was normal. At the age of two weeks after birth, the boy was hospitalized with bleeding from the umbilical wound. In his blood, the levels of the transaminases were high (more than 10-fold). Hyperbilirubinemia with slightly high gamma-glutamyltranspeptidase (GGT) was detected (Table 1). Ultrasound examination of the abdominal cavity showed hepatosplenomegaly and diffuse changes of the liver. The echocardiogram was normal. Brain imaging showed structural changes in the cortical part of the temporal-occipital region of the right hemisphere and the parietal lobe of the left hemisphere with hemorrhagic content. The MS/MS analysis of acylcarnitines and amino acids in DBS and levels of organic acids in urine were normal. The patient had normal bile acid concentration in his urine. Ophthalmological examination revealed retinal angiopathy. The gastroenterologist suggested progressive familial intrahepatic cholestasis, and ATP8B1 gene sequencing was recommended. That test was normal.

**Table 1:** Blood analyses.

	P –IV/2	P-IV/4	P-IV/5
bilirubin common (*N<5 mkM/l)*	141.4	13.7	10.7
bilirubin conjugated (N: *0.5–8.5 mkM/l)*	90.3 (63.9%)	7,9 (57.7%)	6.9 (64.6%)
ALT (N:4–40 E/l)	190.7	59	214
AST (N:4–40 E/l)	588.7	189	557.4
GGT (N:10–60 E/l)	167.8	20.1	30.7
alphafetoprotein (N: <10 ME/ml)	21,450	82.9	270.8
alkaline phosphatase (N:122–469 E/l)	ND	189	205.4
lactate (N:0.9–1.7 mmol/)	3.3	3.6	ND
cholesterol (N:3.2–5.2 mM/l)	4.25	1.24	1.26
urea (N:2.8–7.2 mM/l)	3	3.73	5.55
creatinine (N:54–95 mkM/l)	49.8	33	40
prothrombin index (N:70–130%)	57	45	38
creatinine kinase (N:40–226 E/l)	ND	239	45
LDG (N:225–450 E/l)	ND	489	657
protein (N:65–85 g/l)	96.4	67.6	64.8
albumin (N:35–50 g/l)	44.8	48.6	47.5
Very-long-chain fatty acids (VLCFA)			
C22 (N:25.6–120.6 mmol/ml)	49.4	88.7	ND
C24 (N: 22.6–80 mmol/ml)	54.5	69.6	ND
C26 (N:0.22–2.2 mmol/ml)	15.4	13.2	ND
phytanic acid (N:0–3.11 mg/ml)	0.01	59.42	ND
pristanic acid (N:0,57–0,86 mkmol/l)	1.60	2.11	ND
ratio C26/C22 (N: 0.009–0.018)	0.312	0.149	ND

ALT - alanine aminotransferase; AST - aspartate aminotransferase; GGT – gammaglutamyltranspeptidase; LDG- lactate dehydrogenase; VLCFA - very-long-chain fatty acids.

At the age of seven months, the patient's weight was 5 kg (<3rd centile), height was 57 cm (< 5th centile), and head circumflex was 44 cm (50th centile). The head was large and had a hydrocephalic form with a high forehead and large anterior fontanel (5×6 cm); physical examination showed jaundice, hepatosplenomegaly, and muscular hypotonia. Tendon reflexes were normoactive in the upper limbs, but in the lower limbs, they were present but diminished. The child holds his head tentatively; he demonstrates head lag when maneuver of the traction response, and little forward motion of the head as the child reaches the upright position. He could turn over on his side. He could not sit by himself. He could smile and hum. Gas chromatography–mass spectrometry (GC–MS) analysis revealed the concentration of C26:0 in plasma to be increased up to 15.4 mM/ml (normal 0.22–2.2 mM/ml); the C26/C22 ratio was 0.312 (normal 0.009–0.018), the C24/C22 ratio was 1.102 (normal 0.66–0.88) (Table 1).

**Fig. 2:**
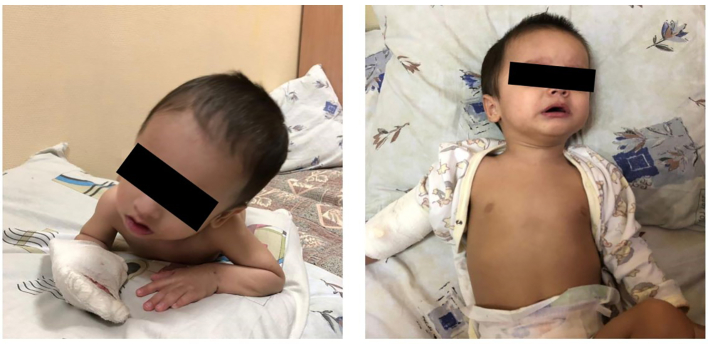
Phenotype of patient IV/4: note the epicanthal folds, high forehead, hypertelorism, broad nasal bridge, and hypoplastic supraorbital ridges.

Patients IV/4 (P–IV/4) and IV/5 (P–IV/5). The patients were 11-month old monozygotic twins. Their mother had four miscarriages and one birth in her anamnesis. Delivery was at the 37th weeks of gestation. The weight and length of the infants were normal. The perinatal period was normal. The first months of their growth and motor development were delayed. At the age of six months, the infants were hospitalized because of bronchitis. In blood tests, levels of transaminases were elevated while the level of GGT was normal and the level of cholesterol was low. Physical examination showed jaundice, hepatosplenomegaly, and muscular hypotonia. An abdominal ultrasound examination showed hepatosplenomegaly and diffuse changes (fibrosis) of the liver. The echocardiograms were normal. MS/MS analysis of acylcarnitines and amino acids in DBS and levels of organic acids and bile acids in urine were normal. At the age of one year, the children's weights were 9.1 kg and 8.9 kg (<10th centile); heights were 76 cm and 77 cm (50th centile); head circumflexes were 48 cm for both (>75th centile). Their phenotypes included macrocrania, high forehead, epicanthal folds, hypertelorism, a broad nasal bridge, and hypoplastic supraorbital ridges (Fig. 2). They could not sit or stand by themselves. They could not speak.

Gas chromatography–mass spectrometry (GC–MS) analysis revealed that the concentrations of C26:0 and phytanic acid in the plasma of patient IV/4 were increased up to 13.2 mM/ml (normal 0.22–2.2 mM/ml) and 59.42 mg/ml (normal 0–3.11 mg/ml), respectively; the C26/C22 ratio was 0.149 (normal 0.009–0.018), the C24/C22 ratio was 0.785 (normal 0.66–0.88) (Table 1).

### 3.2 DNA analyses

**Fig. 3:**
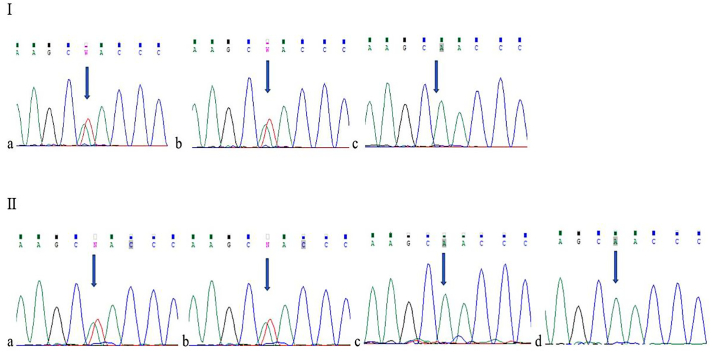
Sanger sequencing of the pedigree. I: patient IV/2 (c) and his mother (a) and father (b). II: patient IV/4 (c), patient IV/5 (d), and their mother (a) and father (b).

The NGS analysis identified only one variant of potential clinical significance in a homozygous state, NM_017929.6:c.347 T>A, p.(Leu116Gln), in the *PEX26* gene. This variant is not present in the Human Gene Mutation Database (HGMD) v. 2020.1 or ClinVar [11]. The population frequency of this variant was unknown [12]. Bioinformatic algorithms classified the variant as probably damaging by PolyPhen-2, SIFT, PROVEAN, and MutationTaster. The mutation found in the affected individuals and family members was confirmed by Sanger sequencing (Fig. 3).

To assess the frequency of the variant in the population of the Republic of Dagestan, DBS samples of 537 newborns from Dagestan were tested for the presence of the c.347 T>A, p.(Leu116Gln), variant in *PEX26* by PCR-RFLP. The screening revealed no carriers of the variant.

## 4 Discussion

Here we describe a family with affected children with ZSD caused by a homozygous mutation in the *PEX26* gene. Initially, we thought that there were two different families with affected children because those families were consulted by different doctors, and the parents informed their doctors that there were no more affected members in their pedigree. Additionally, these families had absolutely different passport data and addresses. But the one thing that they had in common is that they both were of Dargin origin. Dargin is the second-largest ethnic group in the Republic of Dagestan, a federal subject (republic) of Russia, located in the North Caucasus region. Besides Dargins, the people of Dagestan also include a large variety of populations: Avars, Lezgins, Laks, Tabasarans, Chechens, and others [13]. Consanguineous marriages are common there. Since we initially recognized the families to be different, we decided to estimate the frequency of the c.347 T>A, p.(Leu116Gln) mutation in the population of the Republic of Dagestan. We checked 537 dB samples from Dagestan's newborns and found no mutant alleles. Using the Hardy–Weinberg equilibrium we estimated the frequency of the mutant allele in the population of Dagestan to be less than 0.000931 (99% CI: 0.000929–0.000934) and the theoretical prevalence of the disease to be not more than 0.867 (99% CI: 0.862–0.872) per 1,000,000 people, making it extremely rare. Considering the total population size of the Republic of Dagestan (2,910,249 people according to the 2010 All-Russian Population Census), the maximal number of patients should not exceed 2.52 (99% CI, 2.51–2.53) in the whole Republic. Such a rare variant could be a result of the founder effect. Considering the common Dargin origin of the two families, we supposed the families to be distantly related. Indeed, upon gathering additional genealogical information, the two families appeared to have common ancestors in the fifth generation. Summarizing the population genetic findings, it seems that all the cases of ZSD in Dagestan associated with a c.347 T>A, p.(Leu116Gln) variant in *PEX26* are described in the present study. ZSD is a very clinically heterogeneous group of diseases. We know that patients with an early onset of disease have severe symptoms including neurological, hepatic, and other signs. It is known that PBD with an early onset shows severe neurological symptoms. But sometimes it is difficult to find neurological symptoms in infants of the first weeks and months of life, especially in cases when neurological symptoms are less severe than hepatic (as in our patient IV/2). Moreover, hepatic dysfunction with cholestasis and low or slightly elevated GGT are needed to differentiate between various groups of diseases like progressive familial intrahepatic cholestasis (Byler disease) and bile acids synthesis defects because they have different ways of treatment. That is why, VLCFA measurements should be performed in all infants with cholestasis with or without neurological symptoms to early diagnose ZSD rather than isolated phytanic acid analysis which could demonstrate normal level in formula- & breast-fed infants [14].

## 5 Conclusions

We describe three patients affected by ZSD with severe hepatic symptoms and cholestasis caused by a novel homozygous mutation in the *PEX26* gene (NM_017929.6:c.347 T>A, p.(Leu116Gln)). This case report highlights the importance of biochemical screening in infants with hepatic dysfunction and cholestasis.

## Ethical approval and consent to participate

The clinical and molecular genetic study was performed in accordance with the Declaration of Helsinki and was approved by the Institutional Review Board of the Research Centre for Medical Genetics, Moscow, Russia (the approval number 2018-1/3), with written informed consent obtained from each participant and/or their legal representative as appropriate.

## Consent for publication

Written informed consent was obtained from the patients' legal guardians as well as from parents for NBS for publication of this case report, any accompanying images and population studies. A copy of the written consent is available for review by the Editor-in-Chief of this journal.

## Availability of data and supporting materials section

The datasets used and/or analyzed during this study are available from the corresponding author upon reasonable request.

## Funding

The work has been funded by the state assignment of 10.13039/501100012190Ministry of Science and Higher Education of the Russian Federation. The funder had no role in the design of the study; in the collection, analyses, or interpretation of data; in the writing of the manuscript, or in the decision to publish the results.

## Contributions

NS, MK, and AM designed the study. MK performed laboratory experiments and data analysis. ED, TS, NT, and NS collected and interpreted the clinical data. AM did statistical analysis. NSdrafted the manuscript. TS and ED revised the manuscript critically for scientific content. All authors gave scientific and technical input to the study and approved the final version of the manuscript.

## Authors statement

Conceptualization: Natalia A. Semenova, Marina V. Kurkina, Andrey V. Marakhonov, Elena L. Dadali; Data curation: Natalia A. Semenova , Natalia N. Taran, Elena L. Dadali, Tatyana V. Strokova; Formal analysis: Natalia A. Semenova, Marina V. Kurkina, Andrey V. Marakhonov, Elena L. Dadali; Funding acquisition: Natalia A. Semenova Elena L. Dadali; Investigation: Natalia A. Semenova, Andrey V. Marakhonov, Tatyana V. Strokova, Elena L. Dadali; Methodology: Natalia A. Semenova, Elena L. Dadali, Marina V. Kurkina, Andrey V. Marakhonov; Project administration: Natalia A. Semenova, Elena L. Dadali, Tatyana V. Strokova; Resources: Natalia A. Semenova, Elena L. Dadali; Software: Natalia A. Semenova, Andrey V. Marakhonov; Supervision: Elena L. Dadali, Tatyana V. Strokova, Natalia A. Semenova; Validation: all authors; Visualization: Natalia A. Semenova; Writing - original draft: Natalia A. Semenova, Andrey V. Marakhonov, Marina V. Kurkina; Writing - review & editing: all authors.

## Declaration of Competing Interest

The authors declare that they have no competing interests.
